# Autosomal Dominant Hypophosphatemic Rickets Presenting in a Phenotypically Normal Adult Female

**DOI:** 10.1155/2019/8917519

**Published:** 2019-03-04

**Authors:** Hala Mualla, Su Ah Bae, Abid Yaqub

**Affiliations:** ^1^Division of Endocrinology, Diabetes and Metabolism, University of Cincinnati, 231 Albert Sabin Way, Cincinnati, OH 45267-0542, USA; ^2^Cotton O'Neil Diabetes & Endocrinology Clinic, 3520 SW 6th Ave, Topeka, KS 6660, USA

## Abstract

We describe a presentation of Autosomal Dominant Hypophosphatemic Rickets (ADHR) in a 22-year-old female with normal pubertal growth and development and a negative family history in first-degree relatives. The patient presented with a 2-year history of upper and lower extremity proximal muscle pain and weakness and bilateral femoral neck and pubic bone insufficiency fractures. She had a normal serum calcium but a low phosphate as well as 25-hydroxyvitamin D (25(OH)D) levels leading initially to a diagnosis of osteomalacia. Urine phosphate reabsorption was low confirming a phosphate wasting disorder. She had an elevated Fibroblast Growth Factor 23 (FGF23) level. After Tumor-Induced Osteomalacia was ruled out by extensive imaging, she was sent for genetic testing for hereditary rickets which showed a previously reported missense variant in* FGF23*. Subsequently, she found out that her father's maternal aunt and grandfather had ‘bone disorder' and were wheelchair-bound in adulthood. After replenishment of vitamin D, treatment with calcitriol and phosphate leads to complete resolution of patient's symptoms and laboratory abnormalities.

## 1. Introduction

Hypophosphatemic rickets (HR) is a rare genetic disorder characterized by renal phosphate wasting. It is usually transmitted by X-linked inheritance. Autosomal dominant (ADHR) inheritance is relatively uncommon. Here, we report a case of ADHR presenting in 22-year-old female with normal pubertal growth and skeletal phenotype and a negative family history in first-degree relatives. We review the pathophysiology, diagnosis, and treatment of ADHR with emphasis on the clinical presentation in the late-onset form.

## 2. Case Report

A 22-year-old female was referred to our endocrine clinic with 2-year history of gradually progressive proximal muscle pain and weakness involving both upper and lower extremities. Her pediatrician had treated her with prednisone for a period of two months without any improvement. MRI of pelvis and thighs completed before her referral had shown linear hypointense foci in both proximal medial femoral necks and the right ischium consistent with insufficiency fractures. She had a normal childhood development and pubertal growth and at presentation had a height of 154cm and a body weight of 57.1kg. There was no reported family history of rickets, osteomalacia, or any other metabolic bone disease. Her menstrual history was unremarkable.

Past medical history was significant for a diagnosis of carnitine palmitoyl transferase 2 (CPT2) deficiency at the age of 14 which was diagnosed in the context of a work-up for diffuse muscle pain and weakness. At that time, she was evaluated in the Neurology clinic and was found to have low serum carnitine levels, elevated serum alanine, normal lactate, and low pyruvate levels. C16, C18:2, C18:1, and C18 levels were elevated suggesting carnitine palmitoyltransferase II deficiency. However, genetic testing was negative for the S113L variant. Results of other biochemical testing at that time were not available in her medical records. Her symptoms had reportedly completely resolved a few months after she was started on oral carnitine replacement therapy.

Physical exam did not show any apparent kyphosis or scoliosis of spine, vertebral tenderness, or hyperextensibility of joints. There were no lower extremity deformities. She had proximal muscle weakness with 4/5 strength in upper and 3/5 in the lower extremities. There was tenderness in the thighs. Her neurological exam was otherwise normal. She had a waddling gait.

Laboratory work-up revealed the following results: calcium, 9.1 (ref: 8.6-10.2 mg/dl); phosphate level, 2.0 (ref: 2.5-4.5 mg/dl); alkaline phosphatase, 243 (ref: 39-136 Units/L); bone specific alkaline phosphatase, 119.6 (ref: 0-21.3 mg/dl); 25-hydroxy vitamin D, 8.2 (ref: 30-100 ng/ml); and PTH, 145 (ref: 22-84 pg/ml). A bone density scan showed an abnormally low bone density matched for age, gender, and ethnicity with the lowest Z score of -3.3 at the left femoral neck. Vertebral fracture assessment score was within normal limits.

A 24-hour urine collection revealed low calcium of 27.6 (ref: 100-300.0 mg/24 hr) and inappropriately normal urine phosphate excretion of 445 mg/dL (ref: 400.0-1300.0 mg/24 hr). Fractional excretion of phosphate was inappropriately normal at 19% (ref: 15–20%). Renal tubular reabsorption of phosphate (TmP/GFR) was low at 0.50 (ref: 3.18-6.41 for 16- to 25-year-old females). These results suggested renal phosphate wasting as a potential etiology for osteomalacia and we considered Tumor-Induced Osteomalacia (TIO) and Hereditary Hypophosphatemic Rickets (HRR) as diagnostic possibilities. However, in the absence of any family history of rickets and her normal childhood growth and stature, rickets appeared to be less likely and subsequent work-up was directed at ruling out TIO. An Octreotide scan with SPECT imaging came back negative and was followed by F-18 FDG PET/CT scan, which too failed to reveal any tumor. However, an FGF-23 level was found to be elevated at 580 (ref: 44-215 RU/mL). The assay used is a second-generation C-terminal assay that measures both the intact FGF-23 and its C-terminal fragments. At this stage, following the negative localization studies for TIO, the patient was counseled on genetic testing to look for the possibility of hereditary hypophosphatemic rickets. She consented for the genetic testing which revealed a heterozygous known pathogenic missense variant in FGF23: c.527 G>A p. Arg176Gln (R176Q) consistent with a diagnosis of “Autosomal Dominant Hypophosphatemic Rickets”.

Genetic screens for variants in* PHEX* (X-linked Hypophosphatemic Rickets) and* DMP-1* (Autosomal Recessive Hypophosphatemic Rickets 1) were negative. These results were therefore consistent with a diagnosis of Autosomal Dominant Hypophosphatemic Rickets (ADHR) caused by a mutant FGF23. When we informed the patient about the genetic diagnosis, she did some further research regarding her family history and found out that her father's maternal aunt and grandfather had ‘bone disorder' and were wheelchair-bound in early adulthood but her mother remained in good health and had no symptoms

**Figure 1:**
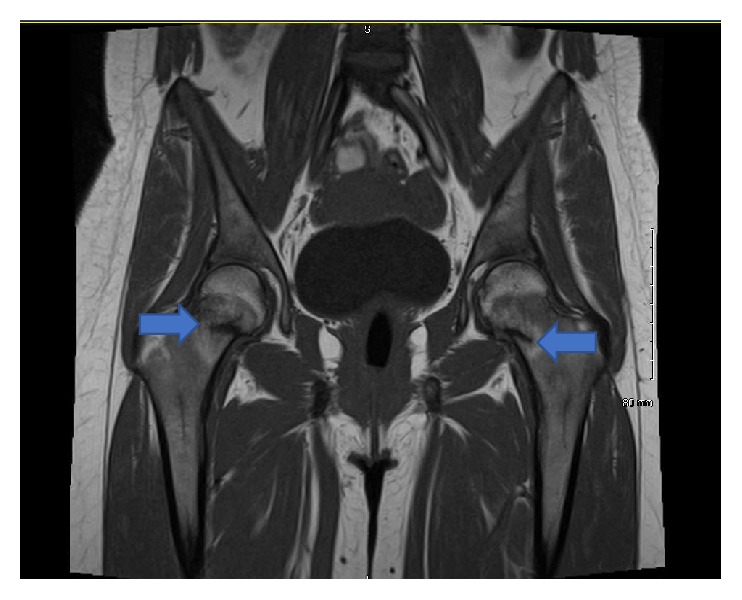
*MRI Pelvis showing bilateral stress insufficiency fractures of the hip, femoral neck*. On the right, the transcervical low signal line extends to the superior lateral cortex and is associated with marrow edema, suggestive of a nondisplaced transcervical fracture.

She was started on Ergocalciferol 50,000 IU weekly and was encouraged to increase her dietary calcium to 1000 mg daily. She was also prescribed Potassium-Sodium-Phosphate 250 mg (8 mmol) 4 times a day. She continued to have pain and discomfort in her inner thighs. She was prescribed Diclofenac and Flexeril for pain. MRI of the pelvis was repeated to follow up on the insufficiency fractures and showed persistent bilateral femoral stress insufficiency fractures (Figure 1). She was referred to orthopedic surgery and underwent pinning of bilateral femoral necks. She had significant improvement in pain and discomfort in the right thigh and groin region but still had some discomfort in the left upper thigh.

Her 25(OH)D levels normalized to 46.2 ng/ml as did her serum phosphate level (2.7 mg/dL) with oral replacement therapy.

**Figure 2:**
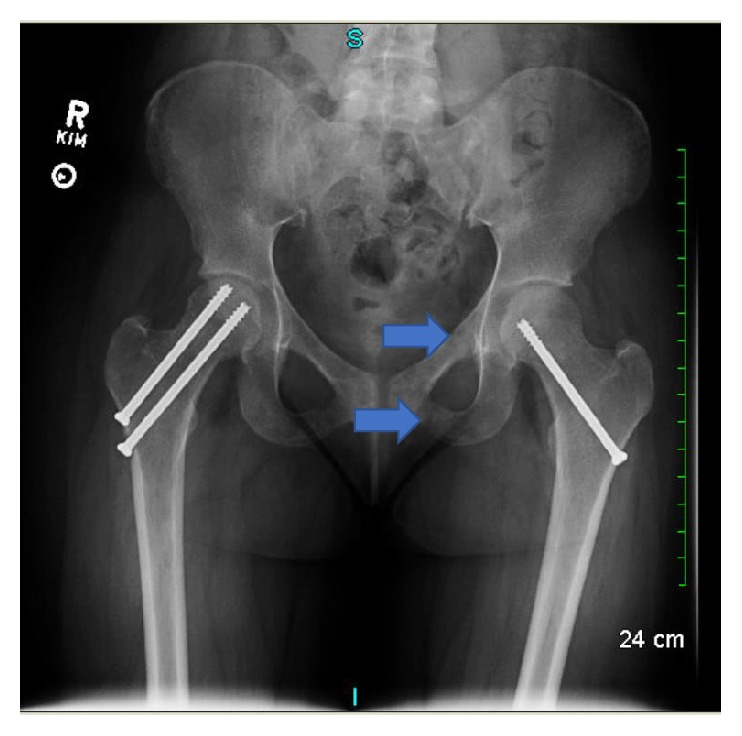
*X-ray of the hips and femurs*. New insufficiency fractures (arrows) were noted. There was interval placement of stabilizing screws for the previous right and left femoral neck insufficiency fractures.

Due to persistent pain in her left groin, she was prescribed narcotic analgesics and underwent a steroid injection of the psoas muscle by orthopedics with no relief. A repeat X-ray of pelvis showed new insufficiency fractures of the left superior and inferior rami (Figure 2).

The patient had difficulty adhering to the regimen of potassium-sodium-phosphate 250 mg (8 mmol) four times daily. Therefore, she was started on calcitriol 0.25 mcg once a day. The dose of calcitriol was gradually increased up to 2 mcg per day. The patient reported significant improvement in her pain after the introduction of calcitriol to her regimen.

Although she missed a few follow-up appointments in the endocrine clinic, she was eventually seen a year later and reported feeling significantly better on the same treatment regimen of calcitriol 2 mcg daily, potassium-sodium-phosphate 250 mg (8 mmol) twice daily, vitamin D3 2000 units daily, and 600 mg of calcium supplement daily. She had complete resolution of her pain and was not on any pain medications. Her physical exam showed normal strength in the upper and lower extremities and no tenderness. Laboratory work-up at that time showed complete resolution of the previous abnormalities: serum phosphate level was 3.3 (ref: 2.5- 4.5 mg/dl); calcium, 9.4 (ref: 8.6-10.2 mg/dl); alkaline phosphatase, 88 (ref: 39-136 units/l); and 25(OH)D, 44.6 (ref: 30-100 ng/ml). A repeat X-ray of pelvis showed resolution of the previous left superior and inferior pubic rami fractures.

The patient subsequently enrolled in a clinical trial of iron supplementation for patients with ADHR at another institution. Correspondence received from that institution showed a hemoglobin 10.1 (ref: 12-15.5 g/dl), hematocrit 31 (ref: 39.4-44.5%), MCV 78 (80-96 fL), platelets 313, 000 (ref: 150,000-400,000/ml), WBC 8.400 (ref: 4,000-11,000 ml), Total Iron Binding Capacity (TIBC) 378 (ref: 240-450 mcg/dl), ferritin 6.4 (ref: 12-300 ng/ml), and percent iron saturation 3% (ref: 25-35%). These results are consistent with iron deficiency anemia. The patient has not followed up with our endocrine clinic since then and we have not been able to reach her despite multiple attempts to obtain any update on her condition following iron supplementation trial.

## 3. Discussion

Hypophosphatemic rickets (HR) is a rare genetic disorder that is characterized by renal phosphate wasting. X-linked inheritance, accounting for about 80% of cases, is the most common form of transmission, with the remaining 20% caused by autosomal dominant (ADHR) and autosomal recessive inheritance [1].

ADHR is caused by missense variants in the* FGF23* encoding Fibroblast Growth Factor 23 (FGF23). These variants disrupt amino acid sequences 176–179 (RXXR), altering the RXXR furin protease recognition site, which protects the mutant FGF23 molecule from proteolysis but does not alter biological activity, leading to increased levels of intact FGF23 molecules, which in turns leads to phosphate wasting at the level of the proximal tubules [2–5].

More than 80% of the filtered load of the phosphate in the kidney is reabsorbed the proximal tubule through the sodium dependent phosphate cotransporters (NPT2a and NPT2c) present on the apical surface of proximal renal tubular cells [6]. FGF23 induces phosphate wasting by reducing expression of the sodium-phosphate cotransporters (NPT2a and NPT2c) on the apical surface of proximal renal tubule cells. FGF23 also suppresses transcription of* CYP27B1*, which encodes the vitamin D 1*α*-hydroxylase, and increases transcription of CYP24A1 which encodes the 24-hydroxylase, resulting in decreased circulating levels of 1,25 (OH)2D which can further exacerbate hypophosphatemia [4, 7–10].

In contrast to X-linked Hypophosphatemic Rickets (XLH), which is the most frequent inherited form of hypophosphatemia [6, 11], characterized by complete penetrance affecting males and females equally, the age at presentation in ADHR can vary between childhood and adulthood due to incomplete penetrance.

In 1997, Econs and McEnery [12] described a large kindred of hypophosphatemic rickets with an inheritance pattern consistent with autosomal dominant transmission. The kindred had 23 affected members and had two subgroups of phenotypes based upon the age of presentation: one group presented in childhood between the ages of 1 and 3 with the typical Ricketic lower extremity bowing and all manifested phosphate wasting, whereas the second group presented with renal phosphate wasting after puberty, between the ages of 14 and 45. The later-onset subjects showed diffuse or localized bone aches, weakness, and insufficiency fractures but did not have the short stature or the typical Ricketic lower extremity deformities or short stature. Their clinical presentation is similar to that for Tumor-Induced Osteomalacia (TIO). In the above-described case series of ADHR, all 9 patients with delayed-onset disease were female and it has been proposed that the postpubertal increases in sex steroid levels may play a role in the delayed-onset or penetrance. Additionally, two patients in the childhood-onset group had spontaneous resolution of their phosphate wasting disorder after puberty. In some instances, the onset of clinically evident disease correlated with a physiological stress such as pregnancy in some of the females in the adult-onset group [12] and a similar case report has also been reported [13].

Following iron supplementation, there was resolution of hypophosphatemia and the need for continued calcitriol and phosphate supplementation, reported in a young girl with ADHR [14]. The girl presented at the age of 26 months with poor growth and severe rickets. She was also found to have iron deficiency without anemia. She required treatment with phosphate supplements and calcitriol twice a day with documented radiological improvement. She relapsed when treatment was stopped around the age of five. She responded to resumption of calcitriol and phosphate supplementation. Around the age of 8, she received high dose iron supplementation, which resulted in significant reduction in both calcitriol and phosphate doses. Around the age of 9, she was able to come off calcitriol and phosphate completely.

It has been proposed that the low iron stores, induced by pregnancy or any other stressors, may lead to disease manifestation or exacerbation later in life. It has been shown that FGF23 expression inversely correlates with iron stores in patients with ADHR as well as in controls. In patients with ADHR, the mutant intact FGF-23 is resistant to cleavage and this leads to exacerbation of disease at times of iron deficiency when there is increased expression of FGF-23. This is not the case in controls, where, despite increase FGF23 expression in the context of iron deficiency, the cleavage process remains active preventing a rise in intact FGF-23 levels [15–17].

Treatment of ADHR involves correction of the abnormalities caused by elevated FGF 23, including low 1,25(OH)2D and serum phosphate, by administration of calcitriol and phosphate. There is limited data on treatment of ADHR as compared to the more common form which is X-linked form of hereditary rickets. The aim of treatment is directed at resolution of symptoms and not just the correction of hypophosphatemia [18].

Our patient, like other reported adult-onset cases, did not have any growth retardation, lower extremity deformity, or any other dental or skeletal abnormality during her childhood or adolescence. Her only reported episode of muscle aches and weakness was in her early adolescence when she was diagnosed with CPTII deficiency biochemically, but with negative genetic testing. We do not have access to her biochemical work-up done at that time. Therefore, it is plausible that she might have had a presentation of ADHR at that time that may have resolved spontaneously. Moreover, iron studies were not checked in our patient prior to her referral or during the work-up at our clinic as the role of iron deficiency and supplementation in management of patients with ADHR was not clearly established at that time. An early diagnosis and management of iron deficiency in our patient would likely have facilitated her management. Our patient did not have full knowledge of her family history, but it appears from her description that ADHR may have had skipped one generation on her paternal side. ADHR has been associated with variable expressivity and penetrance as compared to XLH.

This case highlights the importance of suspecting the diagnosis of hypophosphatemic rickets even in adult patients presenting with musculoskeletal pain, weakness, and hypophosphatemia, renal phosphate wasting, and elevated FGF-23 levels. An in-depth family history is invaluable as it can prevent extensive diagnostic work-up to look for a cause of TIO.

Treatment with calcitriol and phosphate can be remarkably effective in reducing symptoms of pain and weakness as shown in our patient. However, treatment related adverse events can occur necessitating long-term monitoring. Nephrocalcinosis, related to intermittent hypercalcemia and hypercalciuria resulting from high calcitriol dose, and secondary hyperparathyroidism, related to intermittent hypocalcemia on phosphate treatment, have been described. This warrants the need to carefully monitor serum and urinary calcium along with careful adjustment of calcitriol and phosphate dosing to avoid such complications [19, 20]. As shown in the above discussion, treatment of ADHR with iron replacement has been shown to be helpful in some patients leading to complete resolution of pain and biochemical abnormalities without any further need for the conventional treatment with phosphate replacement and calcitriol. It is unclear if our patient was able to get off ADHR treatment regimen upon iron supplementation eventually as she was lost to follow up in our clinic.

Female patients with ADHR or with family history of ADHR need to be followed closely when planning for pregnancy due to possibility of flare-up or worsening of the clinical course. It is unclear if prophylactic iron can be effective in preventing pregnancy-related flare-up of ADHR in such patients.

Another potential future option for patients with ADHR is Burosumab which is a human monoclonal antibody that targets and binds excess Fibroblast Growth Factor 23 (FGF-23) and has been tested in clinical trials in children with X-linked Hypophosphatemic Rickets with success and recently approved by FDA in 2018 for that purpose. Phase 2 trials of Burosumab in children with X-linked hypophosphatemia revealed that it improved renal tubular phosphate reabsorption, serum phosphorus levels, linear growth, and physical function and reduced the pain and the severity of rickets. [21]. However, at present, Burosumab is only approved for children with XLH. Further studies are needed to ascertain if it can be effective in ADHR.

In summary, our case describes a patient with adult-onset ADHR in the absence of any skeletal deformities and highlights the importance of considering this diagnosis in the work-up of patients presenting with musculoskeletal pain, weakness and hypophosphatemia.
